# Pansclerotic Morphea Following COVID-19: A Case Report and Review of Literature on Rheumatologic and Non-rheumatologic Dermatologic Immune-Mediated Disorders Induced by SARS-CoV-2

**DOI:** 10.3389/fmed.2021.728411

**Published:** 2021-10-20

**Authors:** Zahra Lotfi, Anousheh Haghighi, Amirhossein Akbarzadehpasha, Samaneh Mozafarpoor, Azadeh Goodarzi

**Affiliations:** ^1^Department of Dermatology, Faghihi Hospital, Shiraz University of Medical Sciences, Shiraz, Iran; ^2^Department of Dermatology, Rasool Akram Medical Complex, Iran University of Medical Sciences, Tehran, Iran; ^3^Department of Rheumatology, Rasool Akram Medical Complex, Iran University of Medical Sciences, Tehran, Iran; ^4^Department of Internal Medicine, Rasool Akram Medical Complex, Iran University of Medical Sciences, Tehran, Iran; ^5^Department of Dermatology, Skin Diseases and Leishmaniasis Research Center, Isfahan University of Medical Sciences, Isfahan, Iran

**Keywords:** skin disorder, morphea, generalized morphea, dermatology, pansclerotic morphea

## Abstract

While mucocutaneous manifestations of COVID-19 have been frequently reported and added to our knowledge every day during the pandemic, another issue is the COVID-related diseases that can present as intensified lesions of underlying diseases, a new disease, or changes in the behavior of an old lesion. Given that immune system overreaction and cytokine storm are among the most prominent events in COVID-19, the incidence of autoimmune diseases is expected to increase after COVID-19, as confirmed in several reports. To increase the body of knowledge about short- and long-term outcomes of COVID-19 for specialists, it is essential that similar cases be reported and collected for years to come. The present study investigated a case of pansclerotic morphea that rapidly progressed a few weeks after infection with COVID-19 in a 57-year-old woman with no history of any autoimmune skin or rheumatic diseases. She was prescribed outpatient COVID-19 treatment of azithromycin, vitamins D and C, and then quarantined for 2 weeks. The manifestations of the disease were exacerbated at each follow-up and sampling visit at short intervals. This kind of pansclerotic morphea is reported for the first time.

## Key Point

COVID-19-induced autoimmune skin diseases have already been reported. Through reporting a new case of such diseases and a review of the literature, the current article attempts to facilitate the diagnosis of new cases of COVID-induced autoimmune diseases that may occur in the coming years after the pandemic has been contained.

## Introduction

The outbreak of the new SARS-CoV-2 has rapidly spread and infected many people throughout the world since early 2020 (1). Meanwhile, the complications brought by the virus have concerned many people. Given that viruses trigger immune responses, it is predictable that viral diseases cause autoimmune diseases through the viral attack itself or the immune dysregulation due to inflammatory responses. The skin is one of the most important organs that manifest the symptoms and complications of COVID-19 through various types of lesions including exanthematous rashes, urticarial rashes, and mucosal lesions. Since many chronic skin diseases are mediated by immune responses, specialists are in dire need of knowledge about COVID-induced skin diseases. To date, a number of such lesions have been investigated and reported in published articles (2, 3).

The effect of COVID-19 on autoimmune skin diseases can appear as exacerbation of a pre-existing disease (4), changes in manifestations of the disease (5), or causing the disease for the first time. It is worth noting that some of these diseases are exacerbated because patients discontinue immunomodulatory medications, which have been discussed in detail in published guidelines (6).

This is the first case report of pansclerotic morphea (PSM) following COVID-19. In this study, a new case of pansclerotic morphea following COVID-19 infection in a 57-year-old previously healthy woman was studied. After her first symptoms of malaise and stiffness of skin and myalgia, an internal medicine referred her to the rheumatologist. Then a dermatology consult was demanded after some lab tests showing high amounts of ANA and Anti-ds DNA and CRPa. Manifestations of generalized skin stiffness were noted, especially on shins, arms, and abdomen, wherein some areas had the peau d'orange feature. Afterward, a deep biopsy of the skin for further investigations was performed which resulted in sclerodermoid changes. According to clinical examination, the final diagnosis was post-COVID PSM.

## Case Report

A 57-year-old woman with no underlying diseases attended our internal disease clinic on October 15, 2020, presenting with respiratory symptoms, general weakness, and myalgia. Once her PCR test for SARS-CoV-2 was reported positive, she was prescribed outpatient COVID-19 treatment of azithromycin, vitamins D and C, and quarantined for 2 weeks. A retest of that patient on October 28, 2020, was negative, so she resumed her daily functions. During recovery, symptoms of weakness and myalgia persisted, to which arthralgia and arthritis of the ankles and knees were added. Furthermore, difficulty in performing knee flexion impaired the daily functions of the patient. The examinations carried out by the internist ruled out deep vein thrombosis (DVT), hemostasis problems, and heart failure. The lab tests showed high platelet count and ESR, so the physician ordered a complete rheumatology panel. The test results revealed higher than normal ranges for Antinuclear Antibody (ANA), anti-double stranded (anti-ds) DNA, Angiotensin-Converting Enzyme (ACE), and C-reactive protein (CRP). At this stage, the patient was referred to a rheumatologist for further investigations regarding suspected collagen-vascular diseases.

**Figure 1:**
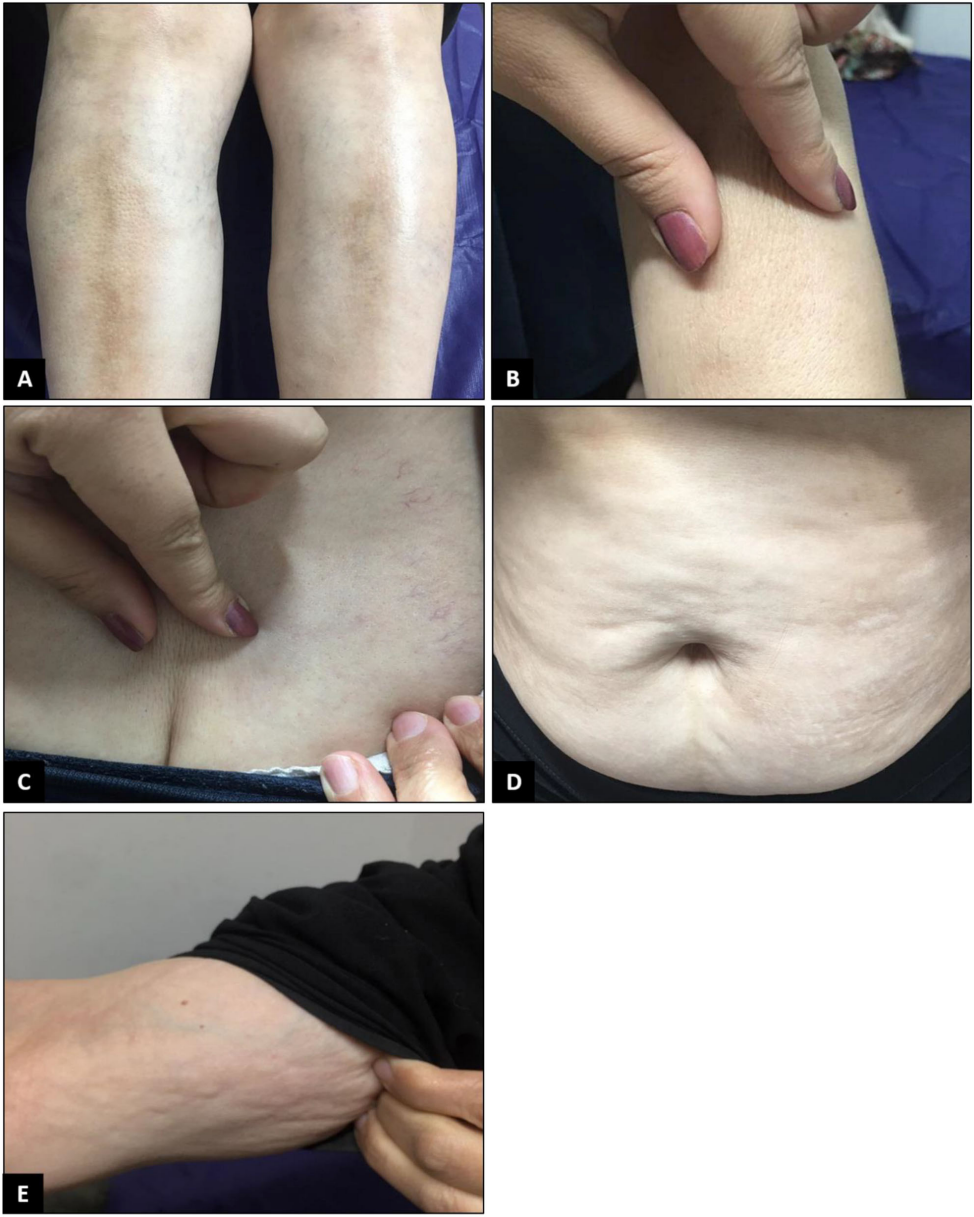
Physical examination revealed the skin had turned shiny and tight. Severe sclerosis was detected on both pretibial regions **(A)**. When touched, the skin felt rather sclerotic and lost the ability to fold compared to normal skin **(B,C)**. Changes in the arm and lower abdomen in favor of morphea were also observed **(D,E)**.

**Figure 2:**
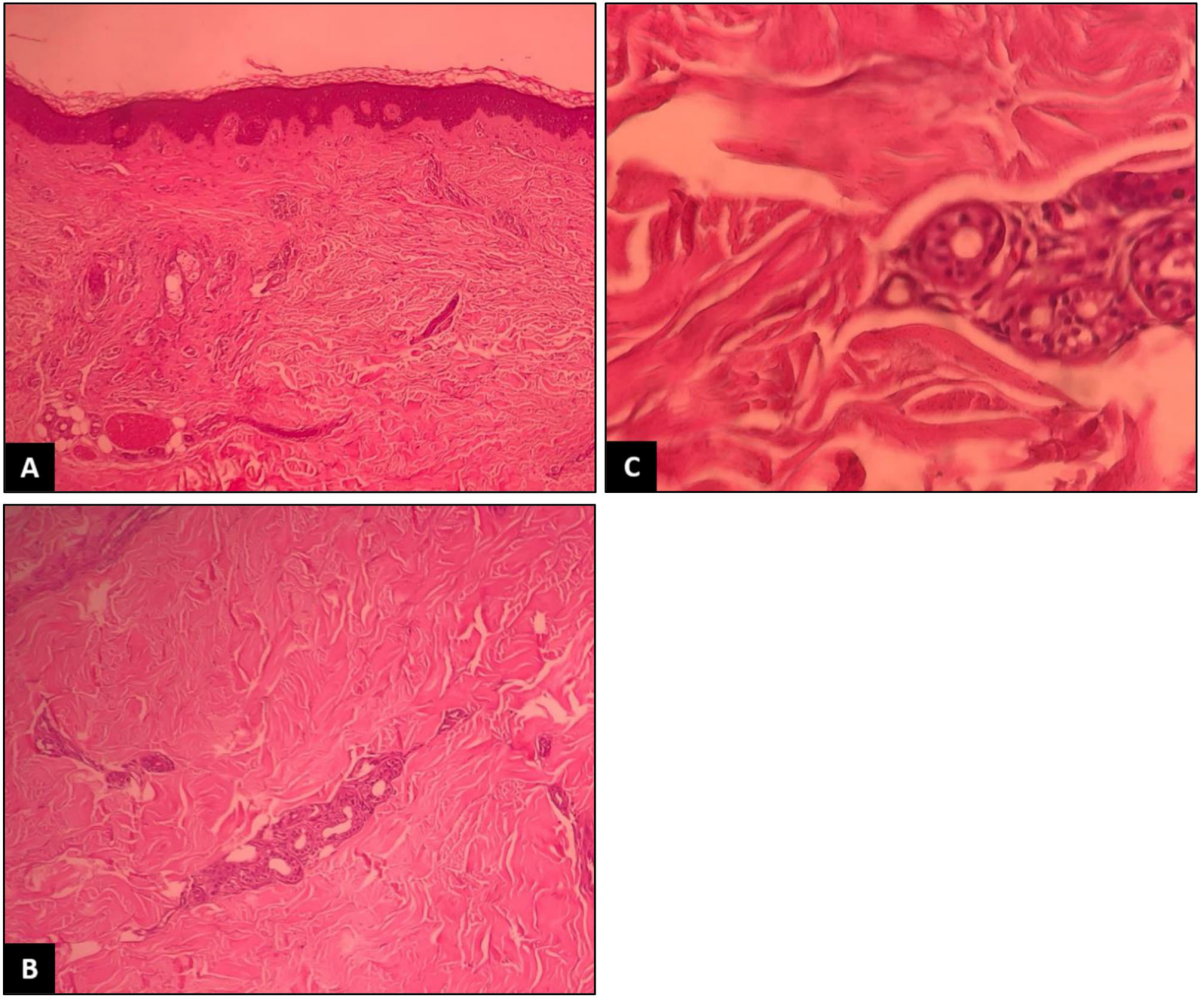
Thickening and hyalinization of connective tissue of deep dermis, subcutaneous fat and muscular fascia, and mucin deposition **(A)**. Atrophy of adnexal structures, increased fibroblastsand dense collagens through the deep dermis **(B)**. Infiltrative changes in the eccrine glands **(C)**. No obvious eosinophilic infiltration was detected.

**Figure 3:**
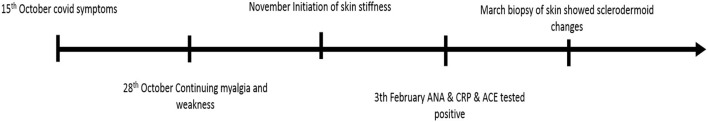
Timeline of events.

The rheumatologist ordered the tests again, which revealed ACE to be higher than the normal range while ANA and anti-ds DNA were negative. Physical examination revealed taut skin and subcutaneous tissue of the left upper limb, in addition to arthritis and arthralgia, so the patient was referred to the dermatology department for a scleroderma work-up. Changes in favor of scleroderma morphea were observed in the first visit of the patient to the dermatology clinic. Physical examination revealed the skin had turned shiny and tight (Figure 1). When touched, the skin felt rather sclerotic and lost the ability to fold compared with normal skin. Severe sclerosis was observed in both pretibial regions. In addition to changes in the arm and lower abdomen in favor of morphea, clinically deep morphea could not be differentiated from eosinophilic fasciitis. Therefore, a deep biopsy was performed on the left pre-tibial and left arm regions which showed changes in favor of sclerodermoid changes and no sign of eosinophilic fasciitis (Figure 2). Re-examination 2 weeks later revealed the exacerbation of previous lesions, newly formed lesions that rapidly spread to the proximal lower limbs and distal upper limbs, and difficult and painful movement of the limb. The pathology report corresponded to scleroderma/morphea in both regions. Treatment initiated with corticosteroids and the patient underwent further examinations while the case report was being written. The timeline of events can be seen in Figure 3.

Given the high levels of CRP and ACE in the lab tests, a CT scan on the lungs was carried out. The CT scan showed a mass in the upper lobe of the right lung, so the patient underwent a needle biopsy, which led to the diagnosis of lung adenocarcinoma. Next, a PET-scan of the lung was performed to assess staging of the adenocarcinoma, and the patient underwent lobectomy of the right lung. Given the patient's underlying conditions, the systemic treatment for morphea was postponed and the patient received only topical medications until the results of the lung cancer assessment were ready. At this stage, the lesions of the patients had stabilized and tissue pain and tenderness reduced. Afterward, lung lobectomy surgery was performed, and the tumor was excised completely. During the follow ups, there were no signs of tumor recurrence. Considering her condition, we preferred to treat her skin condition with topical therapy with corticosteroids and emollients. Then, the patient declared an improvement in pain and stiffness of the skin.

Given the onset of these lesions and their rapid spread immediately after infection with COVID-19, the imbalance of immunomodulatory factors and the activation of the autoimmune response to the virus were considered to have triggered this rapid spread. Lung cancer was accidentally found during the follow-up. Although morphea has been reported as a paraneoplastic syndrome in various types of cancer such as lung small cell carcinoma or breast carcinoma (7–9), it has not been reported as a paraneoplastic phenomenon after adenocarcinoma of the lung. Therefore, its occurrence in this patient can be more attributed to COVID-19 complications. This is the first report of this type of PSM after COVID-19 infection.

## Discussion and Conclusion

Morphea, also known as localized scleroderma, is a chronic autoimmune disease identified by skin inflammation and sclerosis. Scleroderma and morphea are diagnosed with skin sclerosis and have common pathological manifestations. Both diseases present with dermal and subcutaneous sclerosis and no fibroblast proliferation. However, morphea is different from scleroderma in demographic and clinical terms. Unlike scleroderma, involvement of the internal organs is uncommon and the mortality rate is lower in morphea. Different types of morphea are shown in Table 1 (10).

**Table 1:** Categories of different types of morphea.

**Morphea subtype**		**Type**	**Clinical manifestation**
Circumscribed		Superficial	One or more round/oval lesions Histopathological changes limited to the dermis
		Deep	One or more round/oval lesions Histopathological changes involve dermis, subcutaneous tissue, fascia, or muscle
Linear		Trunk/limb	Linear lesions Probably from subcutaneous tissue without the involvement of the dermis May involve muscle or bone
		Head	Progressive hemifacial atrophy (PHA); En coup de saber (ECDS); linear lesions on the face and scalp (with possible involvement of the underlying bone)
Generalized	Coalescent plaque		≥ 4 plaques in at least 2 of the 7 anatomical sites (Head and neck, right/left upper limbs, right/left lower limbs, anterior/posterior trunk) Uniform pattern: interconnected inflammatory plaques in the folds, pelvic girdle, lower abdomen, and proximal thighs. Symmetrical pattern: Peripheral symmetrical plaques around the breast, umbilicus, arm, and legs
	Pansclerotic		Peripheral involvement of large parts of the body surface (without involving the tips of the fingers and toes), including skin, subcutaneous tissue, muscle, and bone. No involvement of internal organs, which is characteristic of scleroderma
Mixed			A mixture of any of the above subtypes (for example: linear—circumscribed)

The generalized morphea is identified by more than four plaques of at least 3 cm that involve two or more anatomical regions. This type of morphea is differentiated from scleroderma by the absence of Raynaud's, sclerodactyly, no facial involvement, no nail fold involvement in capillaroscopy, no visceral involvement, and no specific autoantibodies. Although systemic sclerosis has been reported as a paraneoplastic phenomenon, the association of morphea with cancer has not been demonstrated (11).

Pansclerotic morphea is a type of severe and progressive generalized morphea that deeply spreads into the subcutaneous tissue and invades the muscles, tendons, and bones. The lesions normally appear on the extensor side of the four limbs and trunk, and gradually affect the entire body surface, including the head and neck, causing joint stiffness, deformity, ulceration, and calcification. Squamous cell carcinoma has been reported on the skin lesions of this kind of morphea (12). Disabling PSM of childhood (DPMC) is a rare subtype of juvenile localized scleroderma (JLS) characterized by pansclerosis mainly affecting children under the age of 14. This aggressive disease has a poor prognosis due to the rapid progression of deep musculoskeletal atrophy resulting in cutaneous ulceration and severe joint contractures (13).

Given the stiffness and swelling of the knee in the patient, the above-discussed case was considered to be of PSM type.

There has been much concern about the effect of COVID-19 on the incidence or exacerbation of autoimmune diseases since the outbreak of SARS-CoV-2. Numerous papers have been published about the effects of COVID-19 on the exacerbation of autoimmune diseases. The experience of COVID-19 in people with underlying skin diseases, such as psoriasis, lupus, and rheumatoid arthritis, was documented over time and led to recommendations for modifying the administration of immunomodulatory medications during the pandemic. However, the new cases of these diseases following infection with SARS-CoV-2 when the initial symptoms of COVID-19 abate. Given the high burden of collagen-vascular and chronic skin diseases on the life of the patient, we decided to gather and review articles investigating the incidence of new skin diseases reported after COVID-19 to draw the attention of specialists to this important issue (Tables 2,3). COVID-induced collagen-vascular diseases are presented in Table 2, and other COVID-induced skin diseases in Table 3. It should be noted that COVID-19 vaccination might have some similar effects on immune system responses and cause autoimmune diseases, as there have been some reports to date (14, 15). Therefore, similar reviews of literature and more investigations on that topic are recommended.

**Table 2:** COVID-19-induced collagen-vascular diseases.

**Researchers**	**Treatment measures**	**Tests**	**Skin lesions**	**Patient**	**Row**
			**Timing of lesions**	**Disease**	
Slimani et al. (30)	**Inpatient treatment for COVID-19** = = = = = = = = = = = = = = = = = = = = = = = = Single-dose hydroxychloroquine ————————————— Methylprednisolone	Thrombocytopenia Lymphopenia ↑ PT ↑ D-Dimer ↑ PTT ANA Anti-dsDNA Anticardiolipin Anti-β_2_ Glycoprotein Lupus Anticoagulant ↓ Complement Positive direct coombs test Proteinuria	Papular lesions	23-year-old woman	1
	**No treatment for skin lesions**		13 days after the diagnosis of SARS-COV-2	Systemic lupus erythematosus	
Zamani et al. (31)	**Outpatient treatment of COVID-19** = = = = = = = = = = = = = = = = = = = = = = = = Hydroxychloroquine	Leukopenia Thrombocytopenia ↑ CRP ↑ LDH ↑ Troponin Anti-Ro Anti-La Anti-CCP Anti-dsDNA	Urticaria	43-year-old man	2
	**Treatment for skin lesions** = = = = = = = = = = = = = = = = = = = = = = = = Methylprednisolone pulse ————————————— Hydroxychloroquine ————————————— Prednisolone ————————————— Cyclophosphamide pulse		4 weeks after the diagnosis of SARS-COV-2	Systemic lupus erythematosus	
Bonometti et al. (32)	**Treatment for skin lesion** = = = = = = = = = = = = = = = = = = = = = = = = Single-dose hydroxychloroquine ————————————— Methylprednisolone	Thrombocytopenia ANA Hematuria	Edema, fingertips, and lower limb cyanosis (Vasculitis of fingertips)	85-year-old woman	3
			–	Systemic lupus erythematosus	
Severino et al. (33)	**Treatment for skin lesion** = = = = = = = = = = = = = = = = = = = = = = = = Topical clobetasol	–	White sclerotic lesions with red halo (lilac ring) on the trunk	62-year-old woman	4
			While recovering from SARS-COV-2	Morphea	

**Table 3:** Other COVID-19-induced skin diseases.

**Researchers**	**Treatment measures**	**Tests**	**Ski lesions**	**Patient**	**Row**
			**Timing of lesions**	**Disease**	
Capalbo et al. (34)	**Diagnosis was confirmed by trichoscopy**	–	Some alopecia patches in the beard area	38-year-old man	1
			A month after infection with SARS-COV-2	Alopecia areata	
Rossi et al. (35)	**Diagnosis was confirmed by trichoscopy Treatment for skin lesions** = = = = = = = = = = = = = = = = = = = = = = = = Triamcinolone Acetonide Topical steroids Bimatoprost Vitamin D Probiotics	–	Progressive hair loss with a patchy pattern in the vertex and parietal regions	29-year-old woman	2
			A month after infection with SARS-COV-2	Alopecia areata	
Sgubbi et al. (36)	**Outpatient treatment for COVID-19** = = = = = = = = = = = = = = = = = = = = = = = = Hydroxychloroquine	-	Hair loss with a patchy pattern in the temporoparietal	54-year-old woman	3
	**Diagnosis was confirmed by dermatoscopy Treatment for skin lesions** = = = = = = = = = = = = = = = = = = = = = = = = Topical Clobetasol		Two months after infection with SARS-COV-2	Alopecia areata	
Fivenson et al. (37)	–	–	Rapidly progressive hair loss causing loss of total body hair	56-year-old woman	4
			Two months after infection with SARS-COV-2	Alopecia areata	
Mathieu et al. (38)	**Diagnosis of psoriasis was confirmed by punch biopsy**	–	Blisters on the palms of the hands spreading to the forearms, trunk, and scalp	62-year-old woman	5
			Two weeks after the diagnosis of SARS-COV-2	Pustular psoriasis	
Dadras et al. (39)	**Inpatient treatment for COVID-19** = = = = = = = = = = = = = = = = = = = = = = = = Methylprednisolone pulse	–	Extensive patch and pustular erythematous	60-year-old man	6
	**Treatment for skin lesions** = = = = = = = = = = = = = = = = = = = = = = = = Prednisolone tapering ————————————— Acitretin		26 days after diagnosis of SARS-COV-2	Spreading pustular psoriasis	

It is recommended that reports of new cases of skin diseases be gathered in review articles to help specialists in this field properly diagnose, treat, and manage such diseases.

During the pandemic, the authors especially focused on various skin manifestations of COVID-19 in their research on the subject (16–29).

## Limitation and Strength

Our study had a limitation. We did not long-term follow-up. Because of the pandemic, the authors decided to release the information to be available to researchers as soon as possible. Thus, the diagnosis of lung cancer in between may have affected the results. However, the importance of our study is that it reported a unique and new manifestation, which is the first case of a particular type of autoimmune disease following COVID-19.

## Data Availability Statement

The original contributions presented in the study are included in the article/supplementary material, further inquiries can be directed to the corresponding author/s.

## Ethics Statement

Written informed consent was obtained from the patient for participation in the study and the rights of the subject were protected. To observe ethical principles, the names of the patients were not mentioned in the paper. Written informed consent was obtained from the patient for publication of this case report and any accompanying images.

## Author Contributions

All authors listed have made a substantial, direct and intellectual contribution to the work, and approved it for publication.

## Conflict of Interest

The authors declare that the research was conducted in the absence of any commercial or financial relationships that could be construed as a potential conflict of interest.

## Publisher's Note

All claims expressed in this article are solely those of the authors and do not necessarily represent those of their affiliated organizations, or those of the publisher, the editors and the reviewers. Any product that may be evaluated in this article, or claim that may be made by its manufacturer, is not guaranteed or endorsed by the publisher.
